# Preventing and managing antimicrobial resistance in the African region: A scoping review protocol

**DOI:** 10.1371/journal.pone.0254737

**Published:** 2021-07-14

**Authors:** Chinwe Juliana Iwu-Jaja, Anelisa Jaca, Ishmael Festus Jaja, Portia Jordan, Phelele Bhengu, Chidozie Declan Iwu, Joseph Okeibunor, Humphrey Karamagi, Prosper Tumusiime, Walter Fuller, Ali Ahmed Yahaya, Charles Wiysonge, Laetitia Gahimbare

**Affiliations:** 1 Faculty of Medicine and Health Sciences, Department of Nursing and Midwifery, Stellenbosch University, Cape Town, South Africa; 2 Cochrane South Africa, South African Medical Research Council, Cape Town, South Africa; 3 Faculty of Science and Agriculture, Department of Livestock and Pasture Sciences, University of Fort Hare, Alice, South Africa; 4 Department of Agriculture and Animal Health, University of South Africa, Roodepoort Johannesburg, South Africa; 5 Faculty of Health Sciences, School of Health Systems and Public Health, University of Pretoria, Pretoria, South Africa; 6 World Health Organization Regional Office for Africa, Brazzaville, Congo; 7 Faculty of Medicine and Health Sciences, Department of Global Health, Stellenbosch University, Cape Town, South Africa; 8 School of Public Health and Family Medicine, University of Cape Town, Cape Town, South Africa; Kaohsiung Medical University, TAIWAN

## Abstract

**Introduction:**

Antimicrobial resistance (AMR) constitutes a significant threat to global health and food security, typically associated with high morbidity and mortality rate. The high burden of infectious diseases coupled with the weak health systems in most countries of Africa magnifies the risk of increasing AMR and its consequences thereof. This scoping review will be aimed at mapping the evidence on interventions used to prevent and manage antimicrobial resistance in Africa, guided by the “One Health” concept.

**Methods:**

We will consider interventions targeting multiple sectors such as health care systems, the agricultural and veterinary sectors. The outcomes to be considered include reduction of AMR decreased morbidity and mortality due to infectious diseases, increased awareness for rational use of antimicrobials and reduced antibiotic consumption. We will include all types of studies regardless of study designs conducted within the context of the WHO African region. Studies will be excluded if they are not conducted in Africa and if they are literature reviews, only describing the concept of AMR without mentioning interventions.

We will include studies identified through a comprehensive search of peer-reviewed and grey literature databases. In addition, we will search the reference lists of included studies and relevant reviews. Finally, we plan to do a citation search for included studies. Findings of this review will be narratively synthesized.

## Introduction

Antimicrobial resistance (AMR) is a major global threat to the health of populations, endangering the ability to prevent and cure a wide range of infectious diseases [1–3]. Despite being a global issue, there is a disparate distribution of AMR among countries, with more impact on the developing countries [4–6]. This higher burden on developing countries could attribute to limited access to new antibiotics, increased financial burden, and the inability to pay for second-line antibiotics, which may be more expensive, hence causing worse treatment outcomes. Furthermore, in low-income countries, including Africa, poor Water Sanitation, and Hygiene (WASH) and Infection Control (IPC) measures both at the healthcare and community further increase the occurrence of AMR in microorganisms [7]. Drug resistance has dramatically increased the costs of fighting TB and malaria and slowed the gains against childhood dysentery and pneumonia in Africa [7]. It also threatens the push to treat people living with HIV/AIDS effectively [7].

While the emergence and spread of antimicrobial resistance occur through natural means, there is an increased selection pressure that is mostly caused by excessive and misuse of antibiotics in both human and animal medicine [2,8]. Livestock production is on the increase in developing countries where stringent policies on antibiotic use are not fully in place and implemented [9]. Farmers use large amounts of antimicrobials in livestock including penicillins, β-lactams, fluoroquinolones and aminoglycosides [10]. They also use sub-therapeutic doses in animal feeds to enhance growth, prevent diseases, and increase productivity. The misuse of antibiotics has been associated with high resistance rates in bacteria isolated from food-producing animals to major antimicrobials used in human medicine [11–14]. High resistance rates in bacteria isolated from vegetables and the environment have been reported [11]. Furthermore, the emergence of AMR may be enhanced by substandard or counterfeit antimicrobials [11], little or no regulations on the sale of antimicrobials, inappropriate prescription practices [11], and limitations in public health prevention programs, including immunization, water and sanitation, and sexual health [15].

There is a heightened concern that infections and minor injuries which become treatable might once again be deadly [9]. It has been estimated that by 2050, 10 million deaths will occur due to AMR, with a cumulative economic cost of US$100 trillion [15,16]. The World Health Organization (WHO) indicates that resistance of common bacteria has reached alarming levels in many parts of the world with high-level resistance of *Escherichia coli* and *Klebsiella* spp. to third-generation cephalosporins and carbapenems up to 54% [11,17]. High incidence of antimicrobial resistance significantly reduces the effective treatment of infections, thus increasing complications and severe treatment outcomes, increases hospitalizations, and unnecessary costs on health facilities and patients, and eventually contributed to the end of the antibiotic era [18]. Furthermore, antimicrobial resistance increases the need for more diagnostic tests and additional drugs for treatment [15,19–22]. The imprudent use of antimicrobials can also directly affect the users as well as those not directly exposed to them [23]. Therefore, prudent use of these agents can significantly improve patient safety, ensure the sustainability of antibiotics for as long as possible while limiting catastrophic health care costs.

It should, therefore, be a public health issue of high priority nationally, to ensure the fight against AMR. Measures needed to combat antibiotic resistance have been highlighted such as, adequate awareness of the community on AMR for behaviour change, ensuring that sufficient and essential antimicrobial agents are made available, provision of tools that will rapidly and efficiently diagnose, detect pathogens and carry out their antimicrobial susceptibility, encouraging vigorous WASH and IPC measures such as proper hygiene, immunization, as well as antimicrobial stewardship programs. To combat AMR, the World Health Organization (WHO) developed the global action plan using the “One Health” approach, which was adopted by the 68^th^ World Health Assembly in May 2015 to reduce the widespread rise of AMR [24]. This plan includes a number of well-established interventions such as water, sanitation and hygiene, funding mechanisms, and research to develop new classes of antibiotics, support for and prioritization of new diagnostics, antimicrobials, and vaccines, and education to avoid inappropriate antibiotic use [24]. Antimicrobial stewardship programs have also been formulated, as a way to optimize the use of antimicrobials while preventing the development of resistance and improving patient outcomes [2]. Such programs are made up of several core elements, including new diagnostic technologies or educational programs aimed to avoid excessive use, misuse, or abuse of antibiotics [1,24]. Africa also made their commitment towards promoting AMR surveillance and control by participating and reporting to the Global Antimicrobial Resistance Surveillance System (GLASS) [17].

### The rationale for conducting the review

In Africa, many countries and regions are plagued by weak health systems such as human and infrastructure resource capacities, high burden of infectious diseases, inadequate prevention and control measures, and other social determinants [25]. Hence, there is a high risk of increased AMR and its consequences [11,25]. In fact, AMR has been reported among the common disease pathogens within this region, including HIV, malaria, tuberculosis, typhoid, cholera, meningitis, gonorrhoea, and dysentery [11,25]. Due to the complex nature of AMR, which affects all of society, involves different sectors and is driven by many interconnected factors, single, isolated interventions have limited impact. Hence coordinated actions guided by the “One health” approach are required to minimize the emergence and spread of antimicrobial resistance [1,24].

We searched the literature for reviews on antimicrobial resistance and most reviews identified, described the burden of AMR in Africa as a whole [26], or regionally, like East Africa [27], and other countries within the continent, like Cameroon [11]. Other reviews have also been conducted, focusing on interventions to curb antimicrobial resistance. Most of these reviews have focused on interventions targeting antibiotic prescribing practices by providers, mostly in public health care settings [19,20,28–30]. However, the studies included in these reviews were mostly conducted in high-income and upper-middle-income countries [19,20,28–30], only one in low and middle-income countries [29]. Reviews on interventions for addressing antimicrobial resistance in Africa are scarce, especially those adopting the one health approach [31]. The one health approach in this context comprises of multiple settings and disciplines, including health, animal, and environment sectors, while also taking into account critical determinants such as governance.

Against this backdrop, this scoping review aimed at mapping the existing evidence on interventions used to address antimicrobial resistance in Africa [32]. The scoping review could also be useful in identifying any existing gaps in research. Furthermore, the scoping review could provide more insights for future systematic reviews [32].

### Review question

What is the available evidence on the existing interventions used to prevent and manage antimicrobial resistance in Africa?

### Inclusion criteria

We will be using the Population, Concept, and Context (PCC) framework described by Peters et al. [33] to determine studies that will be eligible for inclusion in this review.

The **Population** of interest will be all the African countries within the African region.

The **concept** comprises of interventions and outcomes. We will include interventions such as training, audit and feedback, advocacy, sensitization, surveillance of AMR, antibiotic stewardship, and any other interventions stated by the authors of included papers. Table 1 shows a detailed list of potential Interventions that countries carry out as guided by the Global Action Plan strategic objectives.

**Table 1:** List of potential interventions that countries carry out as guided by the global action plan strategic objectives.

STRATEGIC OBJECTIVE 1: Improve awareness and understanding of AMR through education and training	STRATEGIC OBJECTIVE 2: Strengthen knowledge and evidence base through AMR surveillance	STRATEGIC OBJECTIVE 3: Reduce the incidence of infection through effective sanitation, hygiene and IPC measures	STRATEGIC OBJECTIVE 4: Optimize the use of antimicrobial medicines in human and animal health
Conduct AMR integrated knowledge, attitude and practice (KAP) behavioural survey to guide training and education	Establish a surveillance system for AMR in human health	Orient/train healthcare workers (HCWs) on the National IPC Guidelines	Develop and enforce legislation on prescriptions for combating AMR
Develop and disseminate a comprehensive communication strategy for AMR for various stakeholders	Establish a surveillance system for AMR in animal health	Strengthen community level prevention	Update key documents (National Medicine Policy, Standard Treatment Guidelines, Essential Medicine List to take into account AMR
Conduct regular public awareness campaigns on AMR	Establish an integrated AMR surveillance system	Strengthen IPC in health care facilities	Establish AMR stewardship programme(s) at National and Health facility levels
Enhance AMR capacity in pre-service institutions (human, animal, environmental, food production and food safety workforce)	Strengthen lab AMR capacity	Strengthen animal health and agricultural biosecurity practices	Establish an antimicrobial prescription monitoring system
Enhance AMR capacity in-service institutions (human, animal, environmental, food production and food safety workforce)	Establish an early warning system to determine risk factors of AMR		Establish a monitoring system for non-prescribed antimicrobials
			Establish a monitoring system for non-prescribed antimicrobials
			Establish/strengthen animal drug regulatory body to address AMR
			Strengthen drug quality control lab
			Strengthen EPA to implement Environmental laws and policies to address AMR

The following outcomes which are guided by the strategic objectives of the global action plan, will be considered:

1. Reduction or containment of AMR
2. Decreased morbidity and mortality to infectious diseases
3. Increased awareness to the rational use of antimicrobials
4. Reduced antimicrobial consumption
5. Any other outcomes described by authors of included studies.

The **context** will be studies conducted in Africa.

#### Types of evidence sources

We will include all types of studies regardless of study designs. These are quantitative studies (e.g., experimental, quasi-experimental, prospective and retrospective cohort, case-control, cross-sectional), observational (e.g., case series, individual case reports, descriptive cross-sectional studies), qualitative studies, mixed-methods studies, narrative reviews [32]. We will also consider systematic reviews and opinion papers in this scoping review. We will include studies conducted in both English and other languages. We will employ the services of a language translator to translate articles written in other languages. No restriction will be placed on the year of publication.

Studies will be excluded if they are not conducted in Africa or if they are literature reviews describing the concept of AMR without interventions. Studies will also be excluded if they estimated the burden of AMR without describing interventions used to address it.

## Materials and methods

**Fig 1:**
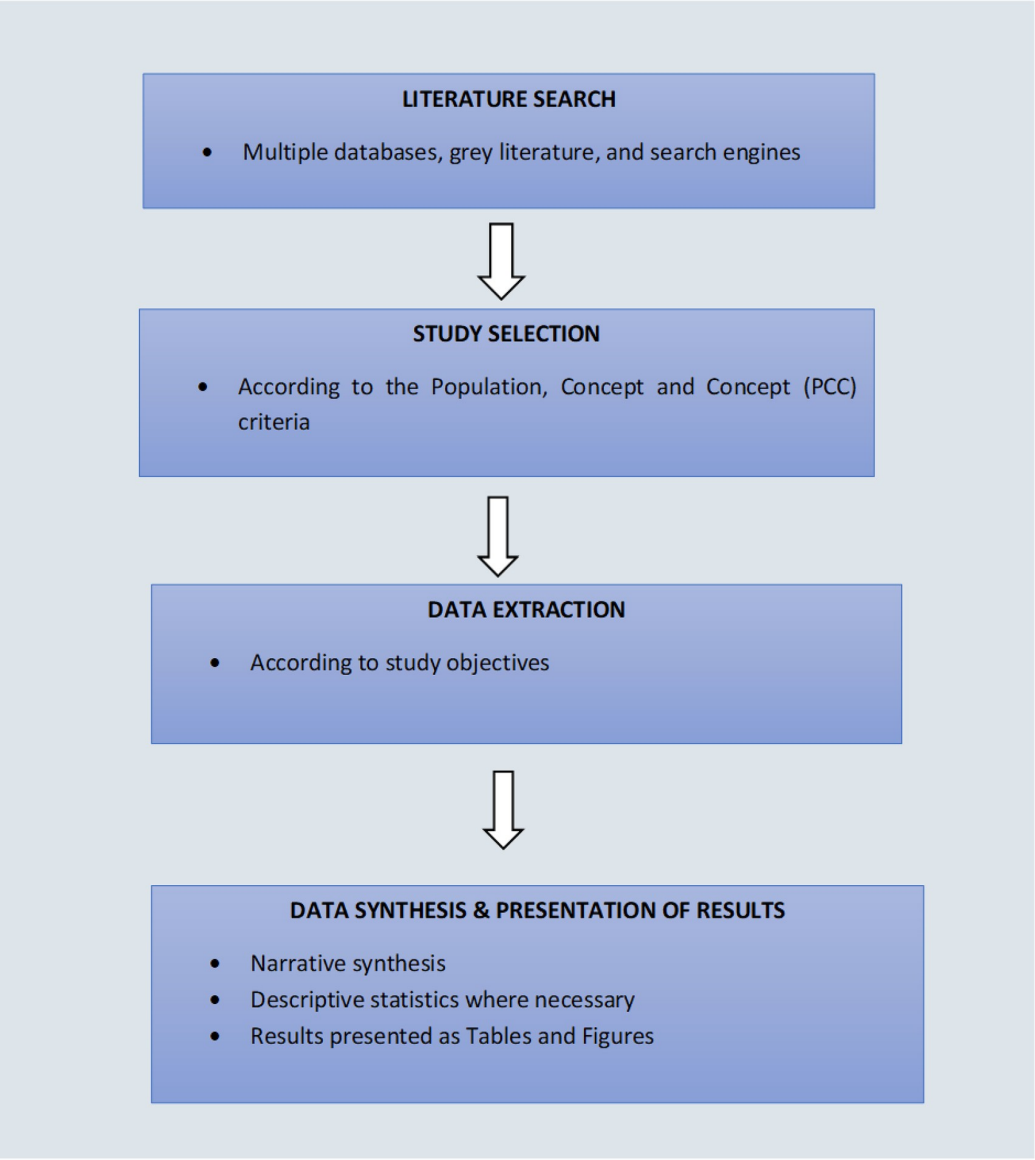
Flow diagram for the scoping review methodology on preventing and managing antimicrobial resistance in the African region.

This scoping review will follow the methodology described by the Joanna Briggs Institute (JBI) as described in the steps below [32] and as shown in Fig 1.

### Search strategy

A comprehensive search strategy will be developed for most databases and grey literature in consultation with a librarian. S1 Appendix shows the search strategy developed in PubMed.

### Information sources

The following databases will be searched: PubMed, Cochrane Central Register of Controlled Trials (CENTRAL), Cumulative Index to Nursing and Allied Health Literature (CINAHL), Scopus, WHO Library Information System (WHOLIS), Web of Science. We will also search the websites of WHO, Centres for Disease and Control (CDC), and Google Scholar. Furthermore, we will search the reference lists of included studies and related systematic reviews.

### Study selection

Following the search, all identified records will be collated and uploaded into Mendeley citation manager (Elsevier, UK) and duplicates removed. Two authors (CJI and AJ) will independently screen the titles and abstracts retrieved from the search for potentially eligible studies. The full texts of these studies will be obtained and further screened for eligibility based on the inclusion and exclusion criteria. These authors will resolve any disagreements regarding eligibility through discussion and consensus. A third author will arbitrate any unresolved differences between the two authors. Reasons for exclusion of full-text papers that do not meet the inclusion criteria will be recorded and reported in the scoping review. The process of study selection will be presented using the Preferred Reporting Items for Systematic Reviews and Meta-Analyses extension for Scoping Reviews (PRISMA-ScR) [34]. Findings from the extracted data will be described narratively.

We intend to engage with relevant stakeholders in the field of antimicrobial resistance for opportunities for knowledge exchange and transfer [35]. These stakeholders could help identify grey literature and provide more insights into the findings of this review.

### Data extraction

Two authors (CJI and AJ) will extract the data independently, from each included study using a piloted data extraction form, adapted from the JBI data collection tool. This form will contain the following information:

1. Authors
2. Year of publication of the article
3. The country where the study was conducted
4. Study design
5. Type of intervention
6. Global Action Plan strategic objective
7. Key findings of the study

Any differences in data extracted will be resolved through discussion and consensus between the two authors. The draft data extraction tool will be modified and revised as necessary during the process of extracting data from each included paper. A third author will be consulted to arbitrate if disagreements persist between the two authors. We plan to contact study investigators to obtain any missing information from included studies. An assessment of methodological limitations or risk of bias of the evidence will not be performed as this is not necessary for scoping reviews [32].

### Data presentation

This will involve a logical and descriptive summary of the results that align with the objective/s of the review and the information contained in the data extraction form. A table summarising characteristics of the included studies and the critical information relevant to the review question will be presented if necessary. This may be further refined during the review.

## Supporting information

S1 Appendix Search strategy developed for PubMed for the scoping review on the prevention and management of antimicrobial resistance in Africa (28th May 2021). (DOCX)
