# Supplementary material for: Preventing and managing antimicrobial resistance in the African region: A scoping review protocol
Source: PLoS One. 2021 Jul 14;16(7):e0254737. doi: 10.1371/journal.pone.0254737 (PMC8279328; doi:10.1371/journal.pone.0254737)
Supplement: S1 Appendix — (DOCX) [file pone.0254737.s001.docx]

**Appendix I: Search strategy developed for PubMed for the scoping review on the prevention and management of antimicrobial resistance in Africa (28th May 2021)**

| **Search #** | **Search Texts and Syntaxes** | **Results** |
| --- | --- | --- |
| #1 | (“Antimicrobial Drug Resistance* OR “Antibiotic Resistance” OR “Drug Resistances, Microbial” OR “Antibiotic Resistance”) | 296,970 |
| #2 | "human health"[Title/Abstract] OR "animal health"[Title/Abstract] OR "environmental health"[Title/Abstract] OR communication[Title/Abstract] OR education[Title/Abstract] OR training[Title/Abstract] OR "capacity building"[Title/Abstract] OR orientation[Title/Abstract] OR campaign[Title/Abstract] OR communication[Title/Abstract] OR "audit feedback"[Title/Abstract] OR surveillance[Title/Abstract] OR sanitation[Title/Abstract] OR hygiene[Title/Abstract] OR "infection prevention control"[Title/Abstract] OR IPC[Title/Abstract] OR "antibiotic stewardship",[Title/Abstract] OR "antimicrobial stewardship"[Title/Abstract] OR "one health"[Title/Abstract] OR legislation[Title/Abstract] OR policy[Title/Abstract] OR guidelines[Title/Abstract] | 591,761 |
| #3 | Search (Africa[Title/Abstract] OR African[Title/Abstract] OR Algeria[Title/Abstract] OR Angola[Title/Abstract] OR Benin[Title/Abstract] OR Botswana[Title/Abstract] OR Burkina Faso[Title/Abstract] OR Burundi[Title/Abstract] OR Cameroon[Title/Abstract] OR “Canary Islands”[Title/Abstract] OR “Cape Verde”[Title/Abstract] OR “Central African Republic”[Title/Abstract] OR Chad[Title/Abstract] OR Comoros[Title/Abstract] OR Congo[Title/Abstract] OR “Democratic Republic of Congo”[Title/Abstract] OR Djibouti[Title/Abstract] OR Egypt[Title/Abstract] OR Eritrea[Title/Abstract] OR Eswatini[Title/Abstract] OR Ethiopia[Title/Abstract] OR Gabon[Title/Abstract] OR Gambia[Title/Abstract] OR Ghana[Title/Abstract] OR Guinea[Title/Abstract] OR “Ivory Coast”[Title/Abstract] OR “Cote d'Ivoire”[Title/Abstract] OR Jamahiriya[Title/Abstract] OR Kenya[Title/Abstract] OR Lesotho[Title/Abstract] OR Liberia[Title/Abstract] OR Libya[Title/Abstract] OR Madagascar[Title/Abstract] OR Malawi[Title/Abstract] OR Mali[Title/Abstract] OR Mauritania[Title/Abstract] OR Mauritius[Title/Abstract] OR Mayotte[Title/Abstract] OR Morocco[Title/Abstract] OR Mozambique[Title/Abstract] OR Namibia[Title/Abstract] OR Niger[Title/Abstract] OR Nigeria[Title/Abstract] OR Principe[Title/Abstract] OR Reunion[Title/Abstract] OR Rwanda[Title/Abstract] OR “Sao Tome”[Title/Abstract] OR Senegal[Title/Abstract] OR Seychelles[Title/Abstract] OR “Sierra Leone”[Title/Abstract] OR Somalia[Title/Abstract] OR “St Helena”[Title/Abstract] OR “sub-Saharan Africa”[Title/Abstract] OR Sudan[Title/Abstract] OR Swaziland[Title/Abstract] OR Tanzania[Title/Abstract] OR Togo[Title/Abstract] OR Tunisia[Title/Abstract] OR Uganda[Title/Abstract] OR “Western Sahara”[Title/Abstract] OR Zaire[Title/Abstract] OR Zambia[Title/Abstract] OR Zimbabwe[Title/Abstract]) | 291 |
| #5 | (#1 AND #2 AND #3) | 324 |
